# Understanding Degradation Dynamics of Azomethine-containing Conjugated Polymers

**DOI:** 10.1021/acs.macromol.4c01168

**Published:** 2024-06-18

**Authors:** Ariane Charland-Martin, Graham S. Collier

**Affiliations:** † Department of Chemistry and Biochemistry, Kennesaw State University, Kennesaw, Georgia 30144, United States; ‡ School of Polymer Science and Engineering, University of Southern Mississippi, Hattiesburg, Mississippi 39406, United States

## Abstract

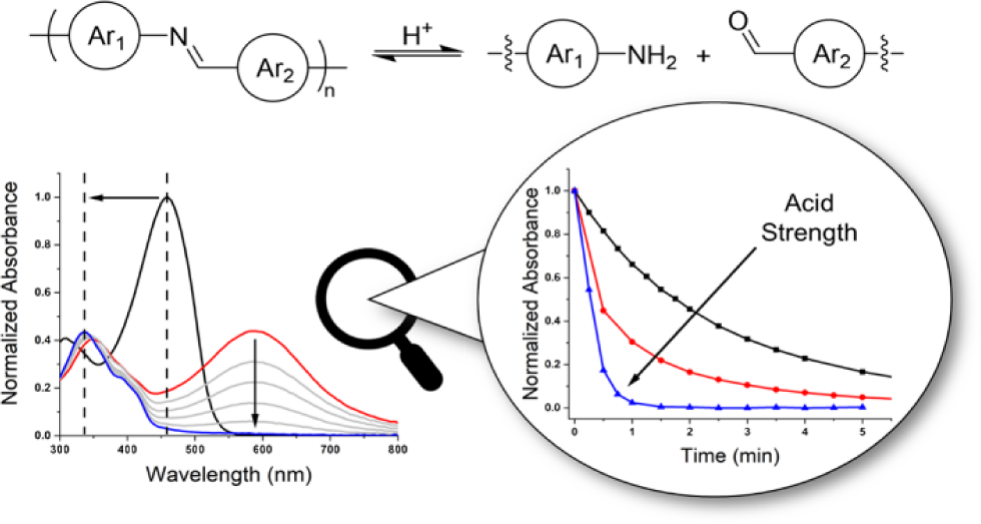

Understanding the influence of chemical environments on the degradation properties of conjugated polymers is an important task for the continued development of sustainable materials with potential utility in biomedical and optoelectronic applications. Azomethine-containing polymers were synthesized via palladium-catalyzed direct arylation polymerization (DArP) and used to study fundamental degradation trends upon exposure to acid. Shifts in the UV–vis absorbance spectra and the appearance/disappearance of aldehyde and imine diagnostic peaks within the ^1^H NMR spectra indicate that the polymers will degrade in the presence of acid. After degradation, the aldehyde starting material was recovered in high yields and was shown to maintain structural integrity when compared with commercial starting materials. Solution-degradation studies found that rates of degradation vary from 5 h to 90 s depending on the choice of solvent or acid used for hydrolysis. Additionally, the polymer was shown to degrade in the presence of perfluoroalkyl substances (PFASs), which makes them potentially useful as PFAS-sensitive sensors. Ultimately, this research provides strategies to control the degradation kinetics of azomethine-containing polymers through the manipulation of environmental factors and guides the continued development of azomethine-based materials.

## Introduction

Electronic devices are traditionally made of inorganic semiconducting materials that are often toxic and require harsh/hazardous conditions to recover raw materials after devices are disposed.^1,2^ The growing consumption of electronics also raises concerns about the environmental impact of discarded inorganic components.^3,4^ With end-of-life considerations in mind, researchers are inspired to focus efforts toward the development of organic semiconductors for transient electronic devices.^5−7^ While conjugated polymers are not inherently benign to the environment, they have the potential to be degradable and are envisioned to leave a smaller ecological footprint compared to inorganic counterparts.^8−10^ Conjugated polymers also offer a number of advantages, including solution processability and tailorable optoelectronic properties, that make them particularly compelling as active-layer materials for flexible devices.^11,12^ Furthermore, these polymers can be modified through the choice of comonomer or side chains to produce materials suited for myriad applications with tailorable solubility and aqueous/biocompatibility.^13−18^ For these reasons, there is a need to gain fundamental insight into the properties of degradable conjugated polymers in order to support the continued development of materials with closed-loop life cycles for electronic devices.

Azomethines have received significant attention as motifs for degradable conjugated polymers.^19−24^ These synthetically simple and environmentally benign materials have been shown to be susceptible to hydrolysis, which makes them suitable candidates for addressing end-of-life considerations of organic electronics.^25,26^ Moreover, azomethines recently have been incorporated into recyclable epoxy thermosets and benzoxazine-containing vitrimers, which demonstrates the impactful nature of imine chemistry beyond conjugated polymers.^27,28^ Under acidic conditions, the heteroatom in the imine linkage (*N*=C) is protonated and the dynamic equilibrium shifts toward the reformation of the aniline and aldehyde starting materials.^29,30^ Many azomethine-containing polymers have been synthesized via acid-catalyzed polycondensations since these protocols offer greener approaches to producing conjugated polymers by avoiding the use of toxic transition metals and producing water as the only byproduct.^31^ For example, the Brochon group synthesized divanillin-based polyazomethines with optical and electronic properties useful for organic light-emitting diodes (OLEDs).^32^ Alternatively, the Bao group synthesized fully degradable fluorene-based polyazomethines to separate semiconducting single-walled carbon nanotubes (s-SWNTs) with low residual polymer impurities.^33^ Our group also recently reported the first example of a degradable azomethine-containing 1,4-dihydropyrrolo[3,2-*b*]pyrrole (DHPP) copolymer synthesized via acid-catalyzed polymerization.^34^ While these examples exploit the benign nature of acid-catalyzed polymerizations, azomethine-containing monomers are also amenable to Pd-catalyzed cross-coupling polymerizations, including Stille and Suzuki reactions, and have been used to synthesize degradable conjugated polymers. Specifically, the Bao and Kim groups have synthesized totally disintegrable polyazomethines and demonstrated their utility as *n*-type and *p*-type semiconductors for transient electronics.^35,36^ Additionally, Tran and coworkers recently reported the first example of degradable azomethine-based conjugated polymers synthesized via direct arylation polymerization (DArP).^37^ The still-increasing range of applications for degradable conjugated polymers emphasizes the importance of polyazomethines and warrants the need to further explore their properties.

Although polyazomethines have been repeatedly shown to degrade via acid hydrolysis of the *N*=C bond, the degradation dynamics have only recently been under investigation. Tran and coworkers reported examples of carotenoid-based polyazomethines that degrade faster when exposed to higher concentrations of hydrochloric acid (HCl) or artificial sunlight.^38^ The Bao group showed that the degradation rate of azomethine-containing diketopyrrolopyrrole (DPP) copolymers can be modulated based on the structure of the side chains. They found that polymers with side chains that have a branching point closer to the polymer backbone, or that are more hydrophilic, tend to degrade faster. The group also observed a solvent dependence on the degradation rate when equal amounts of trifluoroacetic acid (TFA) and water were added to dilute polymer solutions in chloroform (CHCl_3_), toluene, and chlorobenzene.^39^ In another study, Bao and coworkers demonstrated that amorphous polymers with short-range order undergo faster degradation than semicrystalline polymers with long-range order. The increase in degradation rate was attributed to the decreased aggregation and π-stacking interactions of disordered polymers.^40^ Contrastingly, the Skene group has published several examples of azomethine-containing polymers that exhibit resistance to acid hydrolysis.^41−48^ For instance, they demonstrate that some fluorene-containing azomethine copolymers can be reversibly protonated/deprotonated when treated with TFA and triethylamine (TEA), respectively, and do not degrade.^45^ In a separate study, Skene and coworkers studied a polythienylazomethine that underwent reversible acid doping using methanesulfonic acid and TEA without showing any signs of degradation.^49^ More recently, the same group showed that immersing an amido-azomethine film in a TFA/THF solution does not result in electrochemical changes, which provides support that the azomethine film is resistant to hydrolysis.^50^ Combined, these results highlight the effects of chemical environments on the degradation properties of azomethine-containing polymers and motivate further investigation into the variables that influence the degradation rate and hydrolytic stability.

A comprehensive understanding into the influence of environmental factors on the degradation properties of azomethine-containing polymers will be insightful for researchers developing sustainable materials for transient electronics. We hypothesized that an investigation of the degradation kinetics using various solvents and acids would be an effective approach to study and understand the effects of chemical environments on the degradation properties. With these considerations in mind, we synthesized azomethine-containing monomers via acid-catalyzed condensation of brominated aniline and aldehydes. Subsequently, the monomers were copolymerized with 3,3-bis(((2-ethylhexyl)oxy)methyl)-3,4-dihydro-2*H*-thieno[3,4-*b*][1,4]dioxepine (ProDOT) via DArP in order to install imine linkages in the polymer backbone using a simple and environmentally benign approach. The resulting polymer was used to probe degradation trends by monitoring changes in UV–vis absorbance over time in CHCl_3_, toluene, and tetrahydrofuran (THF) after the addition of TFA. The polymer is fully degradable in the presence of organic acids, including TFA, *p*-toluenesulfonic acid (*p*-TSA), and oxalic acid ((COOH)_2_), as well as mineral acids, such as HCl, sulfuric acid (H_2_SO_4_), and phosphoric acid (H_3_PO_4_). Results show that the rate of degradation varies from 5 h to 90 s depending on the solvent or acid used to hydrolyze the polymer. After degradation, the aldehyde starting material was recovered at an 85% yield and with a level of purity that is sufficient for repolymerization. Additionally, the polymer can be degraded with perfluorooctanoic acid (PFOA) and shows potential for detecting perfluoroalkyl substances (PFASs) in the environment. Overall, this study provides fundamental insight into the influence of environmental factors on the degradation dynamics of azomethine-containing polymers that will aid future development of simple materials for degradable/recyclable electronics or controlled release applications.

## Results and Discussion

**Scheme 1:**
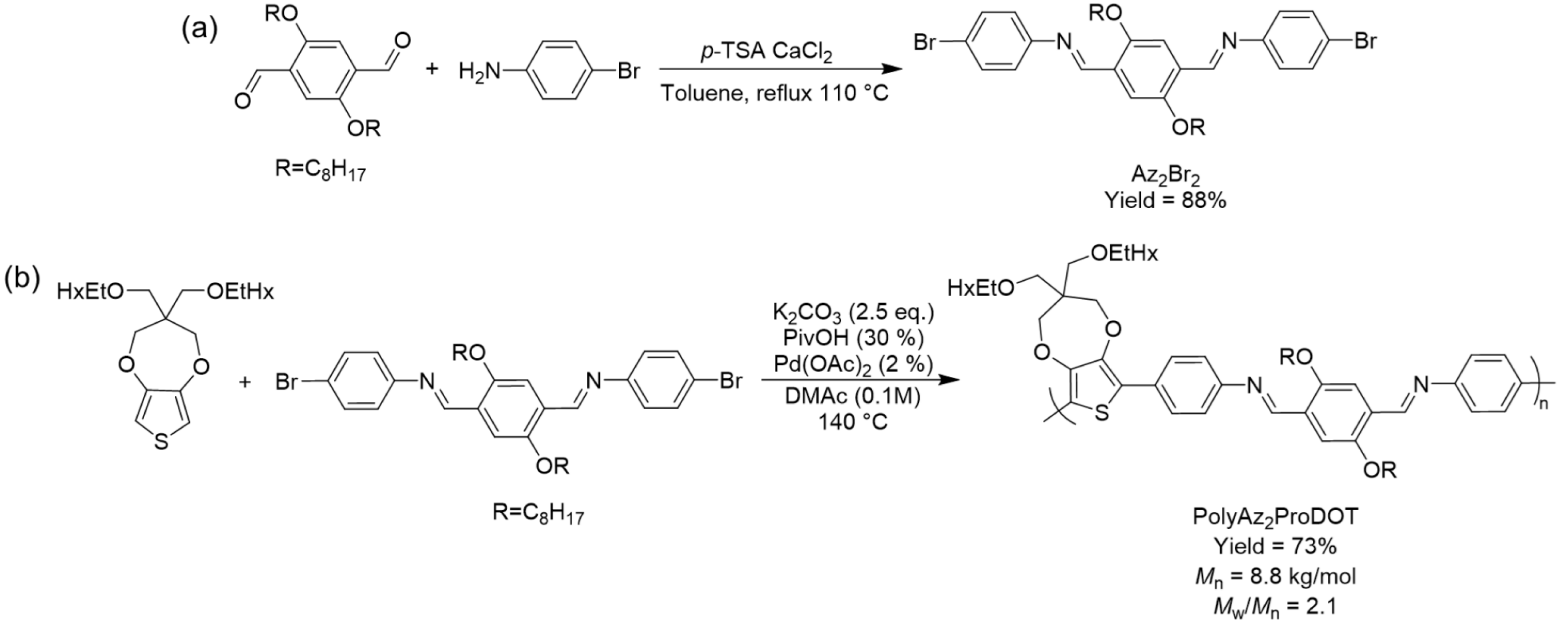
Synthesis of (a) Br_2_Az_2_ via an Acid-catalyzed Condensation and (b) the Reaction Between Br_2_Az_2_ and ProDOT to Synthesize PolyAz_2_ProDOT via DArP

Efforts to study the degradation dynamics of conjugated polymers required the synthesis of azomethine-containing monomers amenable to robust Pd-catalyzed polymerizations by reacting halogenated aniline and aldehydes with an acid catalyst.^33^ Monomers were synthesized with 4-bromoaniline and 4-bromobenzaldehyde (Br_2_Az, Scheme S1) or 2,5-bis(octyloxy)terephthalaldehyde (Br_2_Az_2_, Scheme 1a) using *p*-TSA as the catalyst and the products were purified via recrystallization from chloroform or a 1:1 mixture of hexanes and ethanol (EtOH), respectively. The formation of the resulting imine was confirmed via nuclear magnetic resonance (NMR) spectroscopy by the presence of a singlet at ∼8.5 ppm in ^1^H NMR (Figure S1) and that at ∼160 ppm in ^13^C NMR (Figure S2) for Br_2_Az or at ∼9.0 ppm in ^1^H NMR (Figure S3) and that at ∼155 ppm in ^13^C NMR (Figure S4) for Br_2_Az_2_. Furthermore, the compositional purity of Br_2_Az_2_ was verified using elemental analysis where experimental values were within ±1% of expected values indicating that the monomer was isolated with an appropriate level purity to facilitate polymerizations.

The synthesized azomethine monomers were polymerized via DArP using ProDOT as a comonomer because of the robust electrochemical properties these derivatives possess^51^ and because ProDOT is known to efficiently participate in the catalytic cycle.^52,53^ This protocol offers a greener approach to producing conjugated polymers when compared with traditional methods, such as Stille and Suzuki, by minimizing the number of preparatory steps and toxic byproducts.^54−57^ When combined with the synthesized azomethine-containing monomers, the resulting polymer structure is analogous to early design strategies to attain high gap electrochromic polymers.^58−60^ Furthermore, the ability to design and synthesize ProDOT-containing polymers that can be incorporated into aqueous/biological media^18,61,62^ would make them ideal materials for eventual incorporation into degradable/transient bioelectronic devices. Initial efforts involved polymerizing Br_2_Az with ProDOT, but the resulting material was mostly insoluble and recovered with only a 24% yield (Scheme S3). Motivated to improve solubility, and ultimately facilitate characterization and solution processing, we incorporated octyl ether side chains into the monomeric structure to design Br_2_Az_2_. The copolymerization of Br_2_Az_2_ and ProDOT resulted in an improved yield of 73% for polyAz_2_ProDOT due to the increase in solubility (Scheme 1b). ^1^H NMR indicates that the imine linkage was maintained in the polymer main chain after polymerization as is evident by the retention of the singlet at 9.0 ppm in the spectrum presented in Figure S6. The polymer has a number-average molecular weight (*M*_n_) of 8.8 kg/mol and dispersity (*M*_w_/*M*_n_) of 2.1 as estimated via size-exclusion chromatography (SEC) vs polystyrene (PS) standards when using chlorobenzene as the eluent (Figure S8). Based on the *M*_n_, the degree of polymerization (χ_n_) is estimated to be ∼9 corresponding to ∼36 aromatic rings. Importantly, the χ_n_ of polyAz_2_ProDOT is nearly identical to the indacenodithiophene (IDT) based azomethine polymer synthesized via DArP.^37^ Specifically, the Tran group achieved molecular weights in the range of 10–13 kg/mol with a max yield of 45% while attempting to optimize polymerization conditions, which corresponds to χ_n_ ≈ 9–10 repeat units and ∼40 aromatic rings. They attribute the inability to achieve higher molecular weights to the solubility of the resulting polymers while simultaneously reinforcing the intricacies of optimizing DArP polymerizations. Ultimately, this work presents the second example of azomethine-containing polymers being synthesized via DArP^37^ and a new azomethine-containing polymer that enables investigating the degradation properties of organic semiconductors.

**Figure 1:**
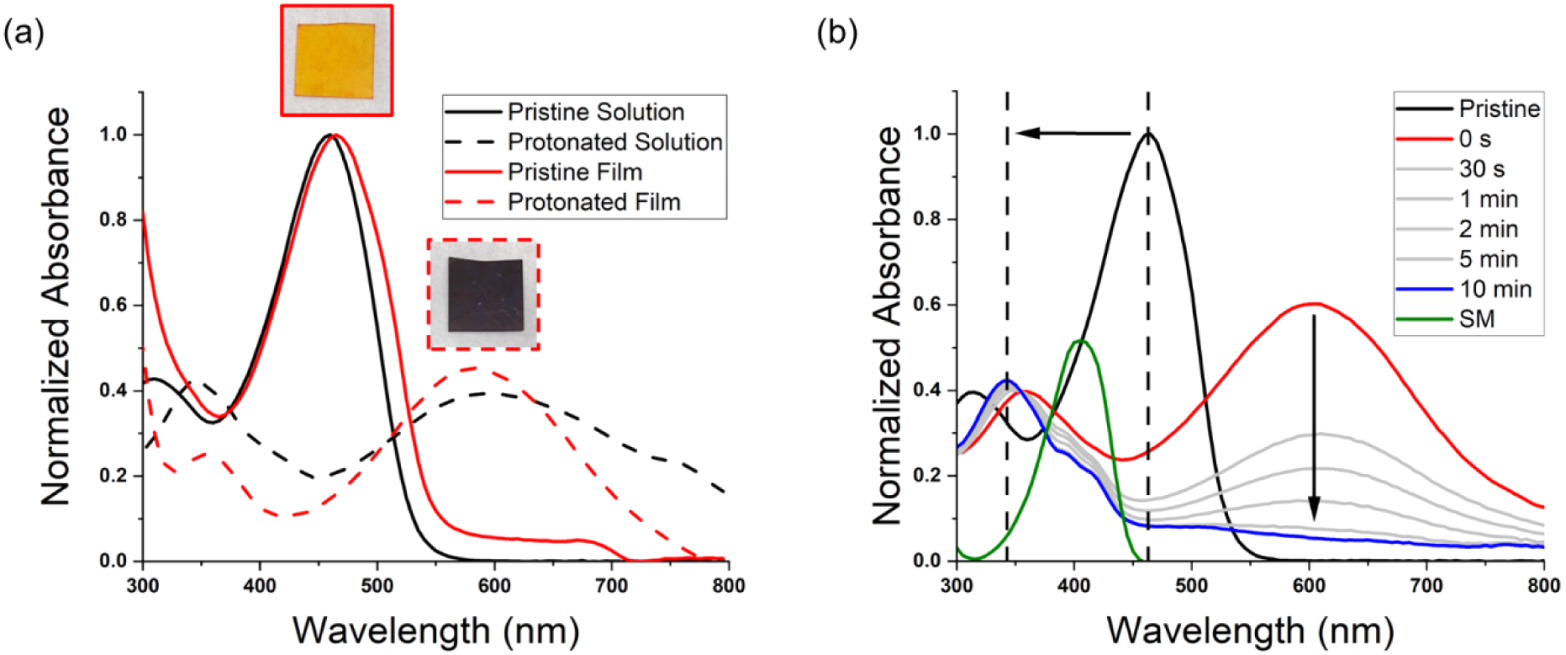
(a) UV–vis absorbance spectra of polyAz_2_ProDOT as a pristine solution in CHCl_3_ (solid black) and immediately after the addition of a 0.4 M TFA/CHCl_3_ solution (dashed black). The red traces correspond to the absorbance spectra of a pristine spin-coated film (solid red) and the same film protonated by TFA vapors (dashed red). (b) UV–vis absorbance spectra as a function of time of a polyAz_2_ProDOT toluene solution after the addition of 50 μL of a 0.4 M TFA/toluene solution. The green trace corresponds to the absorbance spectrum of the 2,5-bis(octyloxy)terephthalaldehyde starting material (SM) in CHCl_3_.

The optical properties of polyAz_2_ProDOT in solution and as thin films were examined via UV–vis absorbance spectroscopy. Although the *M*_n_ of the polymer is considered lower than the threshold for optical properties to be saturated (∼10 kg/mol),^63^ the degree of polymerization is believed to reach the effective conjugated length (ECL). Since χ_n_ ≈ 9, and each repeat unit contains four aromatic rings (three benzene rings and one thiophene ring), the polymer was calculated to have an average of ∼36 rings per chain. Studies have shown that oligothiophenes are estimated to have an ECL of ∼16 rings^64^ and that polyfluorene derivatives can reach the ECL with ∼12 fluorene units or ∼24 aromatic rings^65^ via linear extrapolation of absorption energy vs the inverse of oligomer length plots. Additionally, purine-containing copolymers did not show spectral changes for polymers with more than ∼14 repeat units (3–6 rings per repeat unit).^66^ For these reasons, we are confident to assume that the optical properties measured in this work are an accurate representation of the polymer. As a dilute solution, the polymer displays a featureless absorbance profile with an absorbance maximum (λ_max_) that corresponds to the π–π* transition at ∼460 nm. As a thin film, the λ_max_ exhibits a ∼5 nm red shift accompanied with slight shouldering near ∼500 nm that is ostensibly due to increased ordering of the polymer chains. Upon exposure to TFA, both the polymer solution and film undergo an immediate color change from orange to purple that is accompanied by the appearance of a broad peak from ∼450 to ∼750 nm in the UV–vis absorbance spectrum shown in Figure 1a. The change in color and absorbance is indicative of the imine linkage being protonated and has been observed in other azomethine-containing systems.^21^ As shown in Figure S9, the protonated polymer film reverts to the original orange color after a few hours suggesting that the polymer does not hydrolyze in the solid state and that protonation is reversible in the absence of adequate moisture levels. Additionally, the film also can be oxidatively doped upon exposure to nitrosonium hexafluorophosphate (NOPF_6_) and reduced back to a neutral film in the presence of hydrazine (N_2_H_2_) (Figure S10). This phenomenon could prove useful in designing thin films for write–erase applications.^67^ Furthermore, as shown in Figure S11, differential pulse voltammetry (DPV) experiments reveal the polymer to display an onset of oxidation slightly below 0.6 V vs the ferrocene/ferrocenium (Fc/Fc^+^) redox couple.

The degradation of polyAz_2_ProDOT in solution was further investigated by monitoring the changes in absorbance as a function of time after the addition of TFA. Over the course of 10 min, the protonation peak rapidly diminishes and the solution becomes transmissive. The diminished absorbance and distinct color change (Figure S12) indicate that the imine linkage has successfully cleaved. As shown in Figure 1b, the λ_max_ blueshifts to ∼345 nm and further supports the notion that the polymer has broken down into new chromophores with shorter π-conjugation lengths. The shouldering at ∼400 nm in the absorbance spectrum of the solution at *t* = 10 min overlaps with the λ_max_ of 2,5-bis(octyloxy)terephthalaldehyde at 405 nm (green trace) and suggests that the polymer is breaking down to the aldehyde starting material.

**Figure 2:**
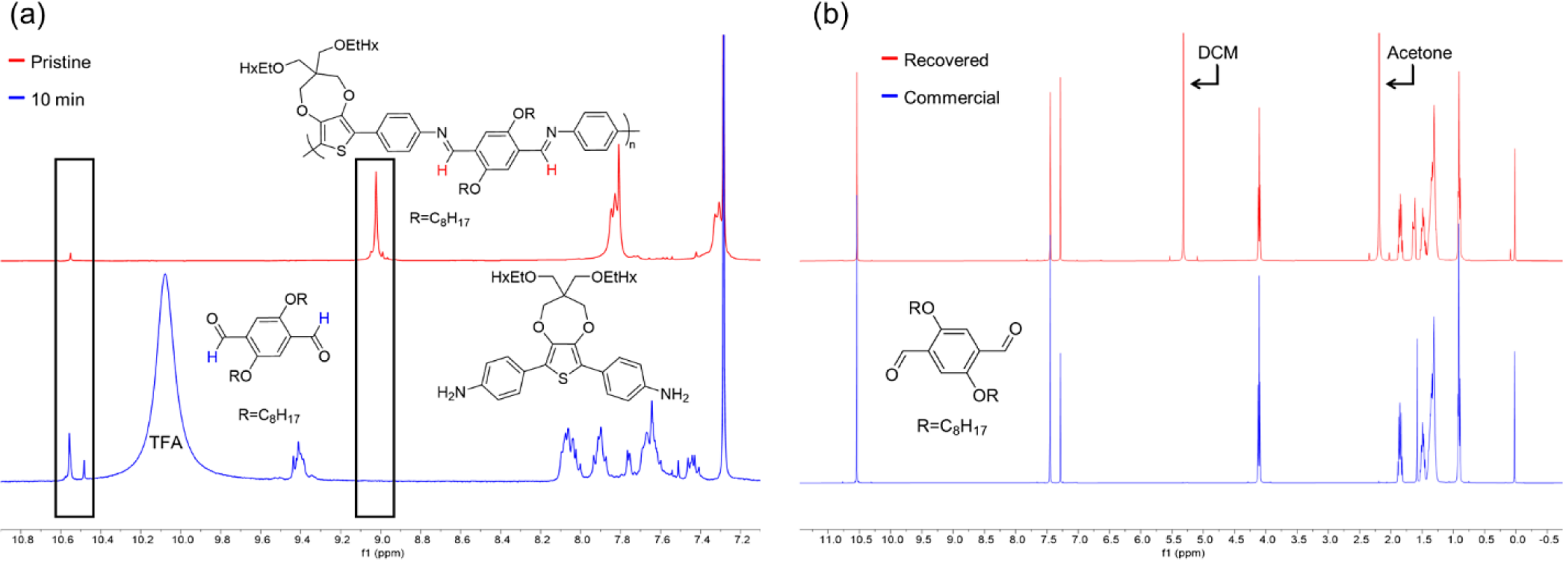
(a) ^1^H NMR (400 MHz, 25 °C, CDCl_3_) of polyAz_2_ProDOT before (red) and 10 min after combination with a 3% v/v TFA-d/CDCl_3_ solution (blue). The box from 8.9 ppm < δ < 9.1 ppm highlights the disappearance of the imine peak with the addition of TFA, and the box from 10.4 ppm < δ < 10.6 ppm highlights the increased intensity of the aldehyde peak. (b) ^1^H NMR of the recovered aldehyde starting material after degradation of polyAz_2_ProDOT with TFA (red) and commercially available 2,5-bis(octyloxy)terephthalaldehyde (blue).

After studying the degradation of polyAz_2_ProDOT via UV–vis absorbance spectroscopy, ^1^H NMR spectroscopy was used to further confirm the cleavage of the *N*=C bond by comparing the spectrum of the pristine polymer with that of the polymer in a 3% v/v deuterated TFA/deuterated chloroform (TFA-*d*/CDCl_3_) solution. As highlighted by the box from 8.9 ppm < δ < 9.1 ppm in Figure 2a, the imine peak present at 9.0 ppm on the pristine polymer spectrum (red trace) is completely absent from the spectrum taken less than 10 min after the sample was exposed to TFA (blue trace). This indicates that the imine linkage has been cleaved and that the polymer is degrading. Furthermore, the increase in intensity of the aldehyde diagnostic peak at ∼10.5 ppm, as shown in the box ∼10.4 ppm < δ < 10.6 ppm, confirms the reformation of the aldehyde starting material and shows promise that the degradation products may be recovered for reuse.

Motivated to investigate the recyclability of polyAz_2_ProDOT, the polymer was degraded in CHCl_3_ at r.t. using TFA as a catalyst and the resulting degradation products were recovered via flash chromatography. The solution transitioned from orange to purple as soon as TFA was added and remained purple for the remainder of the allotted reaction time. After 24 h, the aldehyde starting material 2,5-bis(octyloxy)terephthalaldehyde was recovered with an 85% yield and with a high level of purity. As shown in Figure 2b, the recovered material has identical spectral features to commercially available 2,5-bis(octyloxy)terephthalaldehyde. This study demonstrates another example of starting materials/monomers that can be recovered from azomethine copolymers^7,33,34^ and potentially be utilized to develop active-layer materials with closed-loop life cycles for transient devices.

**Figure 3:**
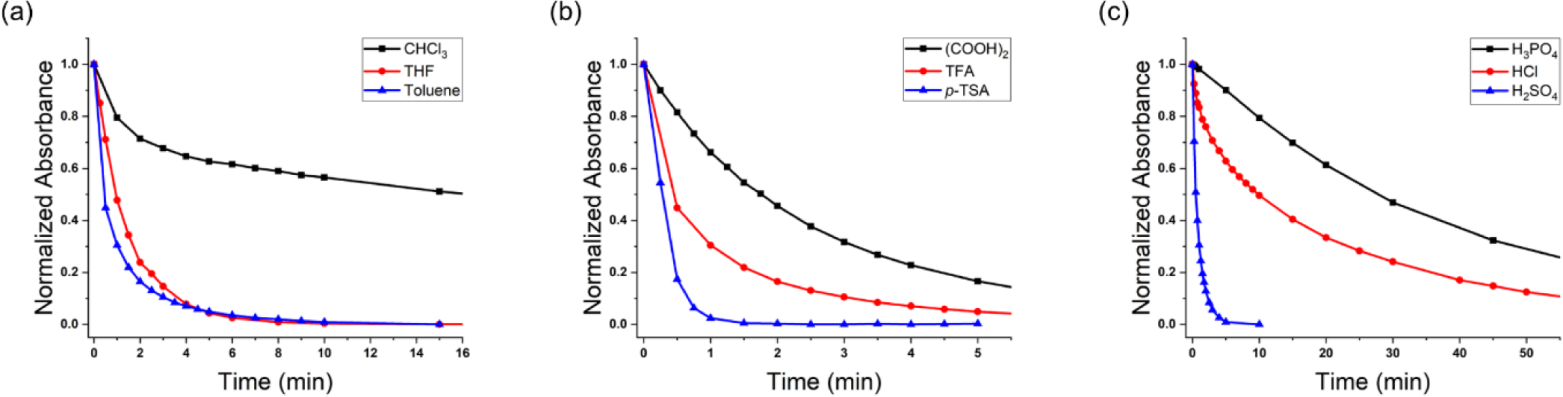
Absorbance at the λ_max_ as a function of time demonstrating the kinetic dependence on (a) solvents, (b) organic acids, and (c) mineral acids.

After confirming the degradability and recyclability of polyAz_2_ProDOT, the rate of degradation and influence of environmental factors on the hydrolysis of polyazomethines were investigated by measuring the changes in absorbance dependent on the solvent or acid. These experiments were designed to establish equal comparison between different chemical environments by first measuring the UV–vis absorbance spectrum of 3.0 mL of a pristine 0.01 mg/mL polyAz_2_ProDOT solution. Subsequently, 50 μL of a 0.4 M acid solution was added to the pristine solution, and the absorbance was measured as a function of time until no more changes in the spectra were observed. The absorbance spectra plotted with respect to time that enabled comparison of degradation kinetics can be found in the Figures S13–S21. Degradation as a function of solvent polarity was studied by monitoring the rate of polymer degradation in solvents with varying dielectric constants (ε) after the addition of TFA. Toluene (ε = 2.38), CHCl_3_ (ε = 4.81), and THF (ε = 7.58) were chosen in this study because of the range of polarities but also because they are solvents commonly used for purifying and processing conjugated polymers. First, as shown in Figure 3a, the polymer degrades significantly slower in CHCl_3_ than in toluene and this result infers a solvent dependence on polymer degradation. This dependence can be justified based on calculations that predict the protonated imine is likely more stable in solvents with higher ε because solvents with higher polarity have a greater ability to insulate/stabilize charges.^68^ However, when THF was used as the solvent, the polymer degrades at a similar rate to toluene and kinetic trends were more difficult to decipher. This difficulty stems from the absence of a protonation peak in the absorbance spectra and the polymer degrading rapidly and directly back to the aldehyde and aniline byproducts. Additionally, THF is known to be highly hygroscopic,^69,70^ and having a greater concentration of water contained in the sample would lead to an increased rate of hydrolysis and deviation from the expected trend. Limited solubility of the polymer in other organic solvents such as ethyl acetate, dimethylformamide, or acetone prevented further investigation into solvent dependence on polymer degradation.

After investigating solvent effects, acids of varying strength were added to dilute polymer solutions in toluene to initiate the imine hydrolysis and relate acid strength to the rate of degradation. The influence of organic acid on the degradation kinetics of polyAz_2_ProDOT was examined using *p*-TSA (p*K*_a_ = −2.8), TFA (p*K*_a_ = 0.2), and (COOH)_2_ (p*K*_a_ = 1.3). While TFA was soluble in toluene, *p*-TSA and (COOH)_2_ required the use of EtOH to make the acidic solutions. As shown in Figure 3b, the polymer fully degraded in ∼90 s with *p*-TSA, ∼10 min with TFA, and ∼30 min with (COOH)_2_. The acid dependence can be rationalized with p*K*_a_ values where acids with a smaller p*K*_a_ (i.e., stronger acids) will facilitate a greater shift in equilibrium toward the formation of the weaker acid (i.e., the protonated imine in this case) and result in a faster rate of degradation. This experiment also used benzoic acid (p*K*_a_ = 4.2), but no change in absorbance was measured; it took up to 4 days for the polymer solution to show any change in color. Considering benzoic acid and protonated imines (p*K*_a_ ≈ 5–7)^71^ are similar in strength, it stands to reason the lack of reactivity under these conditions stems from the acid and protonated polymer quickly reaching equilibrium. This result suggests that the acid-catalyst must have a smaller p*K*_a_ than the iminium ion for degradation to occur. After investigating the effects of organic acids, we were curious as to whether mineral acids had a similar influence on the degradation properties of the polymer and performed further experiments with H_3_PO_4_ (p*K*_a_ = 2.1), HCl (p*K*_a_ = −6.8), and H_2_SO_4_ (p*K*_a_ = −10) in THF. As shown in Figure 3c, the degradation rate of the polymer increases with decreasing p*K*_a_ values (H_3_PO_4_ > HCl > H_2_SO_4_) and follows the same trend as was observed with organic acids. Ultimately, these results provide strategies to control the degradation kinetics of azomethine-containing polymers that can be exploited when designing devices for controlled release applications such as drug-delivery systems.^72−76^

**Scheme 2:**
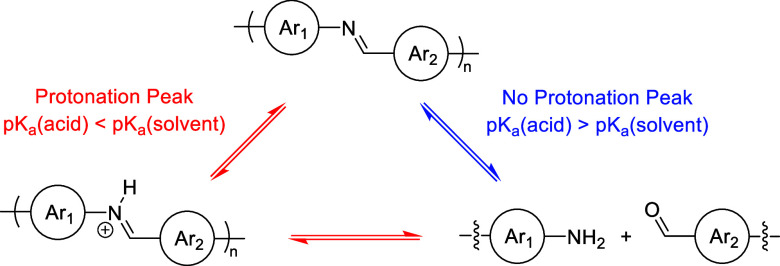
Representative Scheme Demonstrating the Effects of Acid–Solvent Relationships on the Degradation Dynamics of Azomethine-containing Polymers

During analysis of the solution degradation data, the instances where the protonation peak was absent from the absorbance spectra were unexpected. We are not the first to observe this though as Bao and coworkers found it difficult to differentiate between degradation and acid-doping when comparing degradation rates of IDT-based polymers in various solvents.^40^ Upon closer examination, we deduced that the presence/absence of a protonation peak is correlated to the p*K*_a_ of the acid but only when using a solvent, such as THF and EtOH, that has the ability to act as a weak base by accepting a proton that enables the solvent to “compete” in the acid–base reactions. These trends are justified by the reference p*K*_a_ values for protonated THF ((CH_2_)_4_OH^+^) and protonated EtOH (C_2_H_5_OH_2_^+^) being −2.1 and −2.4, respectively.^77^ As illustrated in Scheme 2, when the p*K*_a_ of the acid is less than the p*K*_a_ of the protonated solvent, there is a protonation peak present in the absorbance spectrum (red pathway). The hypothesis is that using a stock solution of a stronger acid, such as *p*-TSA, HCl, and H_2_SO_4_, establishes an equilibrium where the protonated solvent is favored. Upon combination with the polymer solution, and when the iminium ion is a weaker acid than the protonated solvent, protonation of the imine linkage is favored and the positioning of the equilibrium would result in measuring of a protonation peak in the absorbance spectrum. Alternatively, when the p*K*_a_ of the acid is greater than that of the protonated solvent, the protonation peak is then absent from the absorbance spectrum (blue pathway). We theorize that using a weaker acid, such as TFA, (COOH)_2_, and H_3_PO_4_, would diminish the proton exchange reaction between the acid and solvent and prevent the “cascading equilibrium” effect that leads to the protonation peak observed when using stronger acids. This observation is important because the absence of a protonation peak is reflected in the absence of an immediate color change when combining the polymer with acid. Ultimately, having the ability to control this property may prove useful when designing chemical sensors.^78^

**Scheme 3:**
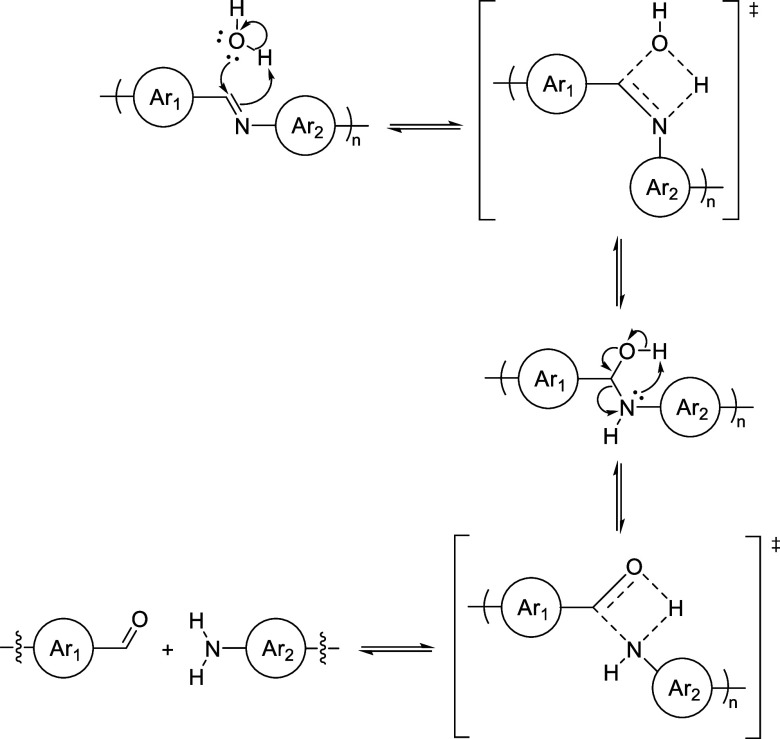
Degradation of Imine Linkage via a Concerted Mechanism

Another plausible explanation for the absence of protonation peaks may be that the degradation process differs mechanistically in certain solvents. The formation of imines is pH dependent in water and hypothesized to undergo a concerted mechanism in organic solvents.^79^ Ostensibly, under certain pH conditions, the degradation may follow a similar mechanism that does not involve the formation of charged intermediates, but rather entails cyclic transition states that would prevent the observation of a protonation peak (Scheme 3). This hypothesis also would justify the accelerated rate of degradation that was observed when using THF as the solvent. THF was hypothesized to have the greatest ability to stabilize the protonated (i.e., charged) polymers because of its higher ε compared to toluene and CHCl_3_, and thus was expected to display a slower rate of degradation. However, the concerted mechanism would bypass the formation of the protonated intermediates that would be stabilized by the polar solvent and likely results in an increased rate of degradation. Furthermore, the concerted mechanism may occur at a faster overall rate due to the labile nature of the tetrahedral hemiaminal (carbinolamine) intermediate.^80^ After formation, the carbinolamine rapidly degrades to the aniline and aldehyde starting materials or reverts to the imine.^81^ This rapid formation/degradation process makes isolation difficult,^82,83^ but the concerted mechanism for the formation of imines has been supported by theoretical calculations.^79^ The plausibility of the concerted mechanism pathway is supported by the results from our acid dependence study in which the degradation rate was recorded to be faster in the absence of a protonation peak although the acid (i.e., TFA) remained constant (see Figure S22).

**Figure 4:**
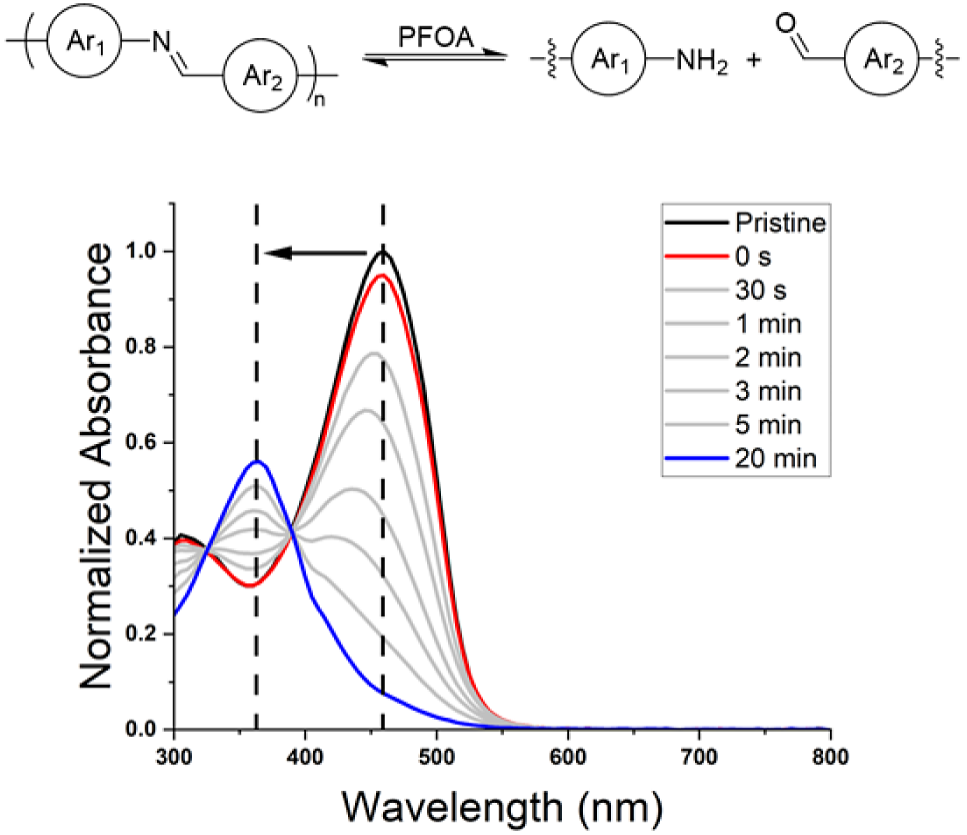
UV–vis absorbance spectra as a function of time of polyAz_2_ProDOT dissolved in THF after the addition of 50 μL of a 0.4 M PFOA/THF solution.

As we were investigating the degradation properties of the polymer in the presence of various acids, we were drawn to the presence of carboxylic acids in PFAS and wondered if polyazomethines were degradable in the presence of this class of molecules. PFASs have been widely commercialized in oil- and water-resistant products and have been found to accumulate in the environment due to their high chemical stability.^84,85^ Growing concerns about the health repercussions associated with exposure to these so-called “forever chemicals” have inspired recent research efforts toward the development of sensors for detecting PFAS in the environment.^86,87^ Current methods of detection require analytical techniques that need to be performed in a laboratory and are not designed for in-field testing or continuous monitoring.^88^ As a result, there is a need for the development of detection techniques that are quick and portable.^89−91^ If the imine linkage is susceptible to hydrolysis when exposed to PFAS, then azomethines could potentially help achieve this goal. With these considerations in mind, 50 μL of a 0.4 M PFOA/THF solution was added to a 3.0 mL sample of 0.01 mg/mL polyAz_2_ProDOT in THF. The polymer degrades in under 20 min as is evident by the blue shift in the λ_max_ from ∼460 nm to ∼365 nm shown in Figure 4. The polymer degrades without the presence of a protonation peak, which suggests the degradation pathway follows the proposed concerted mechanism. In addition to expanding the chemical diversity of acids that degrade polyazomethines, this result has promising implications for the future development of PFAS-detecting sensors. Furthermore, we envision azomethine-containing polymers to be degradable in the presence of other common PFAS, such as PFOS, PFNA, and/or PFHxS, due to the low p*K*_a_ values (∼0–2.5) associated with this class of materials.^92,93^ We acknowledge that the experiments reported here are in organic solvents, but the result inspires the design of aqueous-compatible azomethine-containing polymers that may be useful for future studies. Beyond designing aqueous compatible systems that enable detection in aquatic systems, there will simulatanously be a need to establish structure–property relationships that maximize the limits of detection of known PFAS as well as emerging, nonregulated formulations that will be encountered in the future.^94^

## Conclusion

Azomethines have received significant attention as emerging motifs for degradable materials in numerous device applications. An in-depth understanding of strategies to manipulate the rate of degradation of azomethine-containing polymers is crucial to support the continued development of sustainable materials for transient electronics and controlled release applications. To gain insight into the effects of solvents and acids on the degradation properties of azomethines, azomethine-containing monomers amendable to Pd-catalyzed polymerizations were synthesized and subsequently polymerized via DArP to synthesize an azomethine-containing polymer using environmentally friendly synthetic protocols. Solution degradation studies performed via UV–vis absorbance spectroscopy reveal a dependence on solvent polarity and acid strength. Specifically, the degradation rate of the polymer tends to increase as the polarity of the solvent or p*K*_a_ of the acid used during the hydrolysis of the imine linkage decreases. However, in some instances, the degrading polymer displayed unexpected absorbance profiles in addition to accelerated rates of hydrolysis. We hypothesize that in certain chemical environments, the degradation may follow a concerted mechanism that does not involve the formation of charged intermediates and conceivably occurs at a faster rate. Ultimately, these results confirm that environmental factors can be used to control the rate of degradation of polyazomethines. After degradation, we demonstrate that the aldehyde starting material can be recovered at a high yield and purity level, which allows for starting materials to be recycled. Additional experiments show that azomethine-containing polymers can degrade in the presence of PFAS and could prove useful for detecting PFAS in the environment. Overall, this study provides strategies for tuning the degradation properties of azomethine-containing polymers and expands their utility as sustainable materials with potential application in biomedical and optoelectronic devices.

## Supporting Information Available

Detailed synthetic protocols, ^1^H and ^13^C NMR spectra, size-exclusion chromatography elugrams, supporting spectroscopic analysis, electrochemical data, and photography(PDF)
