# Supplementary material for: Understanding Degradation Dynamics of Azomethine-containing Conjugated Polymers
Source: Macromolecules. 2024 Jun 18;57(13):6146–55. doi: 10.1021/acs.macromol.4c01168 (PMC11238594; doi:10.1021/acs.macromol.4c01168)
Supplement: Supplementary file 1 — ma4c01168_si_001.pdf [file ma4c01168_si_001.pdf]

Supporting Information

Understanding Degradation Dynamics of Azomethine-Containing Conjugated Polymers

Ariane Charland-Martin[1] and Graham S. Collier[1,2]*

[1]Department of Chemistry and Biochemistry, Kennesaw State University, Kennesaw, GA, 30144, United States

[2]School of Polymer Science and Engineering, University of Southern Mississippi, Hattiesburg, MS, 39406, United States

Materials and Method

All chemicals were purchased from commercial sources and used as received unless otherwise noted. 3,3-Bis(((2-ethylhexyl)oxy)methyl)-3,4-dihydro-2*H*-thieno[3,4-*b*][1,4]dioxepine (ProDOT) was synthesized following a published protocol.[1] Solvents were obtained from a Pure Process Technology GC-SPS-7 Glass Contour 800 L Solvent Purification System stored under argon (Ar).Toluene and *N*,*N*−dimethylacetamide (DMAc) were degassed with Ar for ~ 15 min before use. Chromatography was performed with a Teledyne Isco CombliFlash R_f 150 set at a flow rate of 18 mL/min using a 6:4 hexane:dichloromethane (Hx:DCM) mobile phase and a 12 g RediSep Silver flash column packed with 60 Å silica gel (200-400 mesh). ^{1}H and ^{13}C NMR spectra were recorded using a Bruker Ascend 400 MHz NMR spectrometer with a nominal sample concentration of 10-15 mg/mL in $CDCl_3$. Peaks were referenced to the residual $CHCl_3$ peak (^{1}H: δ = 7.26 ppm, ^{13}C: δ = 77.23 ppm). Solution and film absorbance spectra were collected on an Agilent Technologies Cary 60 UV-Vis spectrophotometer scanning from 300 to 800 nm. Solution UV-vis was performed with a nominal sample concentration of 0.01 mg/mL in $CHCl_3$, toluene, or THF. Polymer films were spin-coated onto

2.54 x 2.54 cm glass slide from a 15-20 mg/mL solution in $CHCl_3$ using an Ossila spin coater set at 1400 rpm for 30 s. Prior to spin-coating, the glass sides were cleaned via untrasonication for 5 min in actetone then isopropyl alcohol (IPA) before being dried with compressed air. Differential pulse voltammetry (DPV) was measured using an EG&G Princeton Applied Research Potentiostat/Galvanostat Model 263A under CorrWare control in a three-electrode cell configuration, using indium tin oxide (ITO) coated glass (Delta Technologies, 7 × 50 × 0.7 mm, SiO_2 passivated, sheet resistance (R_s) = 8-12 Ω/sq) as the working electrode, an Ag/AgCl reference electrode (calibrated vs. the Fc/Fc^+ redox couple, $E_{1/2}$ = 363.5 mV), and a Pt flag as the counter electrode, with a step time of 0.1 s, step size of 2 mV, and amplitude of 25 mV. An electrolyte solution of 0.1 M tetra-*n*-butylammonium hexafluorophosphate ($TBAPF_6$, 98%) in anhydrous acetonitrile (ACN) was used for all electrochemical measurements. The polymer film was deposited onto an ITO-coated glass slide from a 3 mg/mL $CHCl_3$ solution via spray-coating using an Iwata airbrush at 20 psi. The ITO slide was cleaned by sequential sonication in acetone and IPA before being dried with compressed air. Polymer molecular weight is reported relative to polystyrene (PS) standards and was estimated via size-exclusion chromatography (SEC) using a Tosoh EcoSEC high temperature GPC operated at 130 °C using 1,2,4-trichlorobenzene (TCB) as the eluent with a flow rate of 1 mL/min. Samples were prepared by dissolving 3 mg of polymer in 1 mL of TCB and stirring at 120 °C for 3 h before filtering through a 0.45 μm PTFE membrane filter. Elemental analyses were performed by Atlantic Microlab Inc. All pictures are presented without manipulation except for cropping.

Procedures for Synthesizing Azomethine Monomers via Acid-Catalyzed Condensation

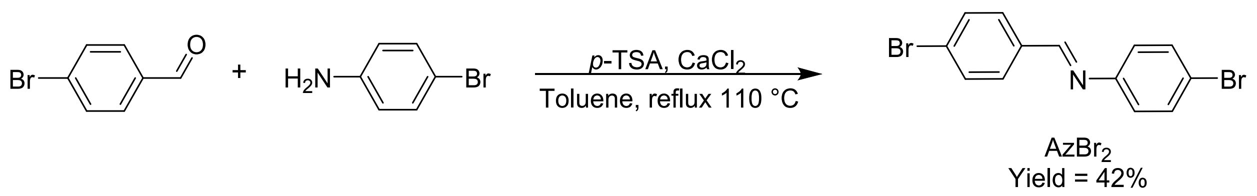

Scheme S1. Synthesis of Br_2Az via acid-catalyzed condensation.

All azomethine-containing monomers were synthesized following a literature procedure reported by Lei et al.[2] with slight modifications. Br_2Az was synthesized by dissolving 4-bromobenzaldeyde (529.2 mg, 2.86 mmol), 4-bromoaniline (492.0 mg, 1 eq.), *p*-TSA (27.2 mg, 5 mol%), and anhydrous $CaCl_2$ (400 mg, ~ 1.2 eq.) in toluene (100 mL, 0.1 M) in a 250-mL 3-neck round bottom flask. The flask was rendered inert via vacuum/refill cycles (3x) with Ar and the reaction mixture was stirred with a magnetic stir bar for 48 h at reflux in an oil bath thermostatted at 110 °C. After the allotted reaction time, dry K_2CO_3 (40 mg, 10 mol%) was added and the reaction was stirred for 30 min while refluxing. The reaction mixture was cooled to r.t. before being filtered and washed with toluene to remove insoluble salts and drying agents. The solvent was evaporated via rotary evaporation and the crude product was dried under vacuum. The crude product was recrystallized from chloroform and collected *via* vacuum filtration.

***N*,1-bis(4-bromophenyl)methanimine (Br_2Az):** White solid, 405.0 mg (42%). ^1H NMR (400 MHz, 25 ºC, $CDCl_3$), δ: 7.11 (d, 2H), 7.53 (d, 2H), 7.65 (d, 2H), 7.79 (d, 2H), 8.41 (s, 1H). ^{13}C NMR (400 MHz, 25 ºC, $CDCl_3$), δ: 119.66, 122.56, 126.23, 130.22, 132.13, 132.28, 134.86, 150.60, 159.30.

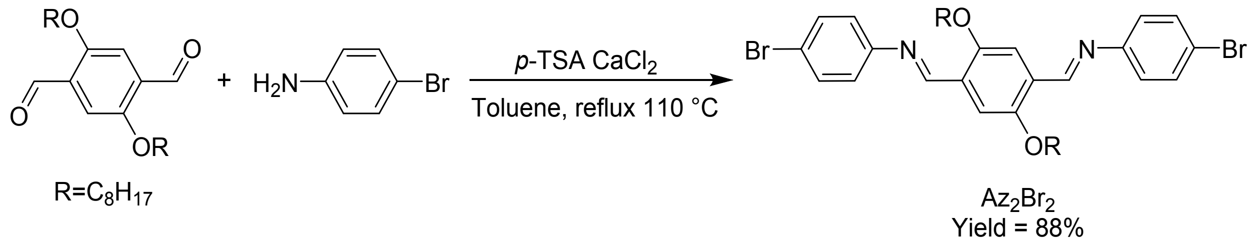

Scheme S2. Synthesis of Br_2Az_2 via acid-catalyzed condensation.

Br_2Az_2 was synthesized by dissolving 2,5-bis(octyloxy)terephthalaldehyde (558.5 mg, 1.43 mmol), 4-bromoanline (541.2 mg, 2.2 eq.), *p*-TSA (12.3 mg, 5 mol%), and anhydrous $CaCl_2$ (350 mg, 2.2 eq.) in toluene (50 mL, 0.1 M) in a 100-mL 3-neck round bottom flask. The flask was rendered inert via vacuum/refill cycles (3x) with Ar and the reaction mixture was stirred with a magnetic stir bar for 48 h at reflux in an oil bath thermostatted at 110 °C. After the allotted reaction time, dry K_2CO_3 (20 mg, 10 mol%) was added and the reaction was stirred for 30 min while refluxing. The reaction mixture was cooled to r.t. before being filtered and washed with toluene to remove insoluble salts and drying agents. The solvent was evaporated via rotary evaporation and the crude product was dried under vacuum. The crude product was recrystallized from a 1:1 mixture of hexanes and ethanol (EtOH) and collected via vacuum filtration.

1,1'-(2,5-bis(octyloxy)-1,4-phenylene)bis(*N*-(4-bromophenyl)methanimine) (Br_2Az_2): Yellow solid, 875.6 mg (88%). ^{1}H NMR (400 MHz, 25 ºC, $CDCl_3$), δ: 0.90 (t, 7H), 1.30 (m, 21H), 1.50 (qu, 5H), 1.85 (qu, 5H), 4.14 (t, 4H), 7.14 (d, 4H), 7.54 (d, 4H), 7.75 (s, 2H), 8.98 (s, 2H). ^{13}C NMR (400 MHz, 25 ºC, $CDCl_3$), δ: 14.10, 22.66, 26.14, 29.25, 31.79, 69.26, 110.60, 119.44, 122.80, 128.26, 132.22, 151.45, 153.50, 156.32. Anal. calc'd for $C_{36}H_{46}Br_2N_2O_2$: C 61.90; H 6.64; N 4.01 Actual: C 61.77; H 6.62; N 3.85.

General Procedure for Synthesizing Azomethine-co-ProDOT Polymers via Direct Arylation Polymerization (DArP)

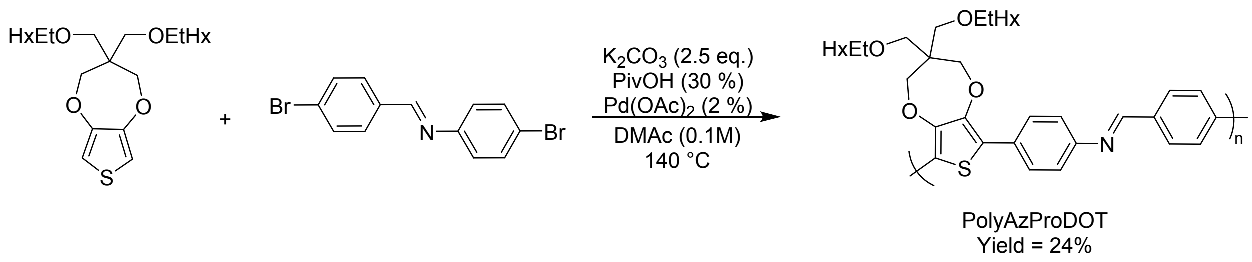

Scheme S3. Synthesis of polyAzProDOT via DArP.

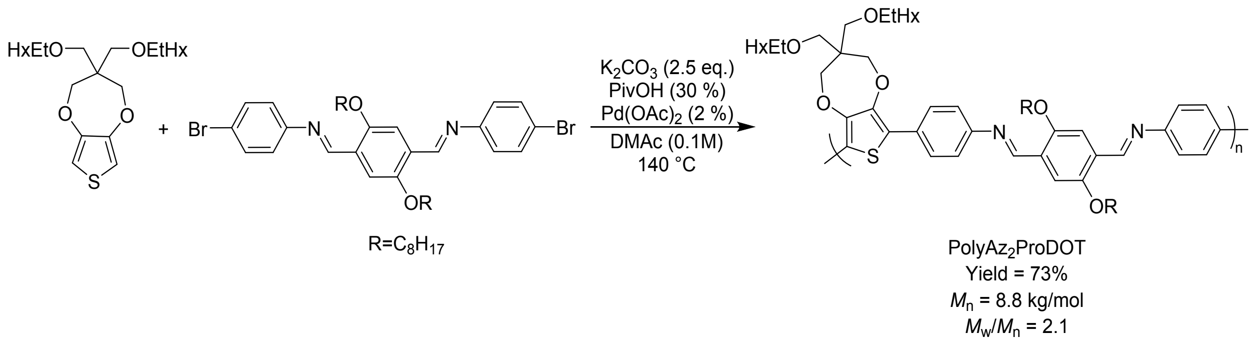

Scheme S4. Synthesis of polyAz$_2$ProDOT via DArP.

Azomethine-co-ProDOT polymers were synthesized by combining Br_2Az (125.2 mg, 0.4 mmol) or Br_2Az_2 (279.4 mg, 0.4 mmol), K_2CO_3 (138.2 mg, 2.5 eq.), PivOH (12.3 mg, 30 mol%), and $Pd(AOc)_2$ (1.8 mg, 2 mol%) in a 10-mL Schlenk flask. The flask was rendered inert via vacuum/refill cycles (3x) with Ar and anhydrous dimethylacetamide (DMAc) was degassed with Ar for 15 minutes. ProDOT (176.3 mg, 1 eq.) was weighted out in a clean vial and DMAc (4.0 mL, 0.1 M) was used to transfer ProDOT into the reaction flask. The reaction was lowered into an oil bath pre-heated to 140 °C and stirred overnight. After stirring for 24 h, the resulting mixture was cooled to r.t. and precipitated in methanol (MeOH). The precipitate was collected into a cellulose thimble and purified via Soxhlet extraction. The product was washed with MeOH and acetone before being extracted with chloroform. The solution was concentrated using rotary

evaporation before being precipitated into MeOH. The polymer was collected on a polytetrafluoroethylene (PTFE) membrane filter via vacuum filtration and dried under vacuum overnight.

PolyAzProDOT: Orange solid, 55.0 mg (24%). ^{1}H NMR (400 MHz, 25 ºC, $CDCl_3$), δ: 0.93 (br s, 42H), 1.32 (m, 56H), 3.36 (s, 15H), 3.62 (s, 12H), 4.25 (d, 10H), 7.11 (br s, 3H), 7.65 (br s, 8H), 7.82 (br d, 4H), 7.91 (br d, 4H), 8.52 (s, 1H).

PolyAz$_2$ProDOT: Red solid, 287.4 mg (73%). M_n = 8.8 kg/mol, M_w/M_n = 2.1. ^{1}H NMR (400 MHz, 25 ºC, $CDCl_3$), δ: 0.93 (t, 25H), 1.32 (m, 45H), 1.54 (m, 7H), 1.88 (qu, 5H), 3.36 (d, 5H), 3.63 (s, 5H), 4.19 (t, 5H), 4.24 (s, 3H), 7.32 (d, 4H), 7.81 (s, 2H), 7.84 (d, 4H), 9.02 (s, 2H). Anal. calc'd for $C_{61}H_{90}N_2O_6S$: C 74.80; H 9.26; N 2.86; S 3.27 Actual: C 75.24; H 9.22; N 2.90; S 3.27.

NMR Spectroscopy

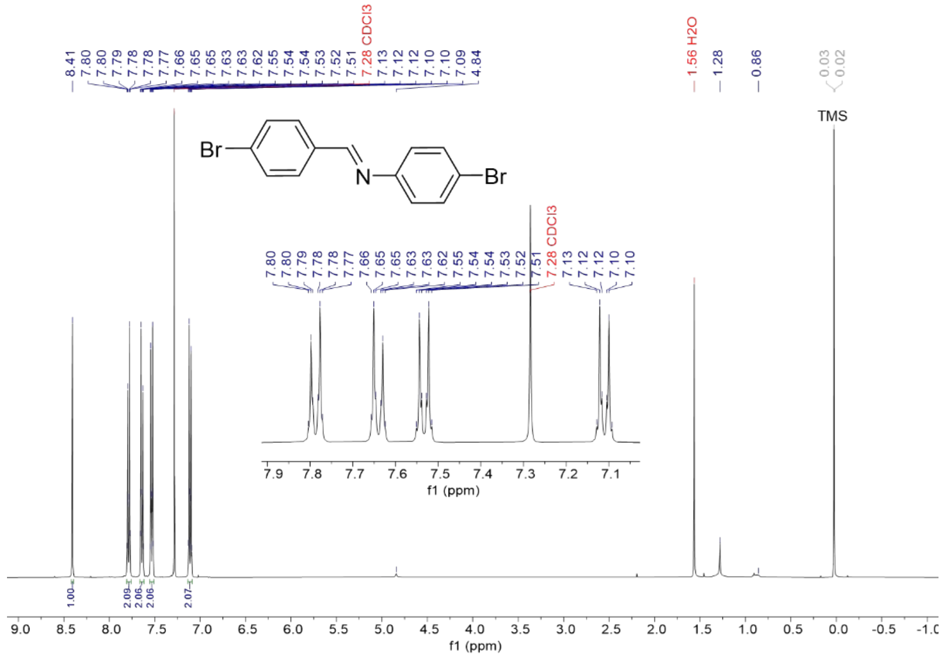

Figure S1. ^{1}H NMR (400 MHz, 25 ºC, $CDCl_3$) of Br_2Az.

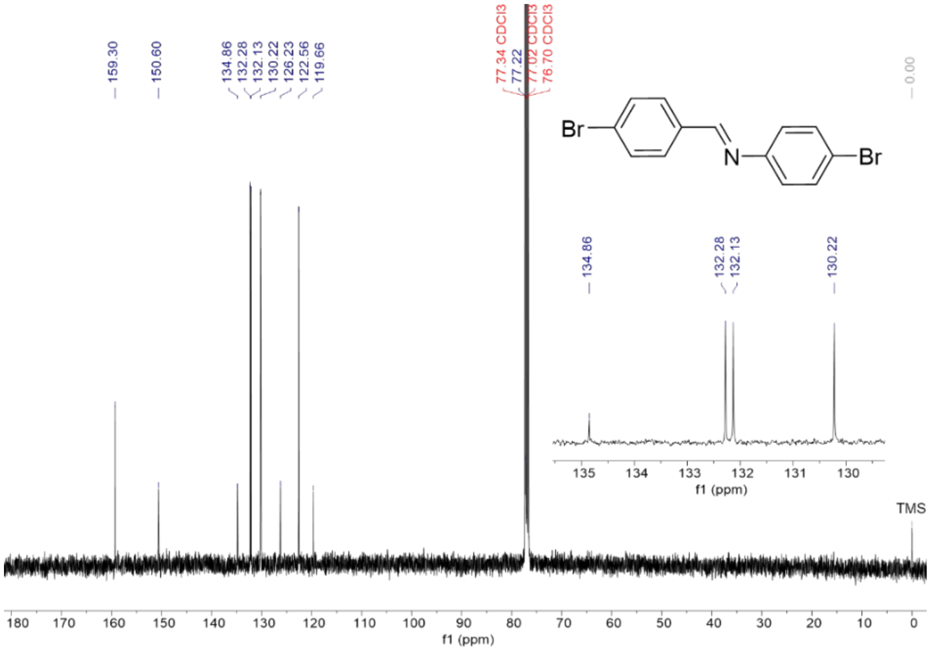

Figure S2. ^{13}C NMR (400 MHz, 25 ºC, $CDCl_3$) of Br_2Az.

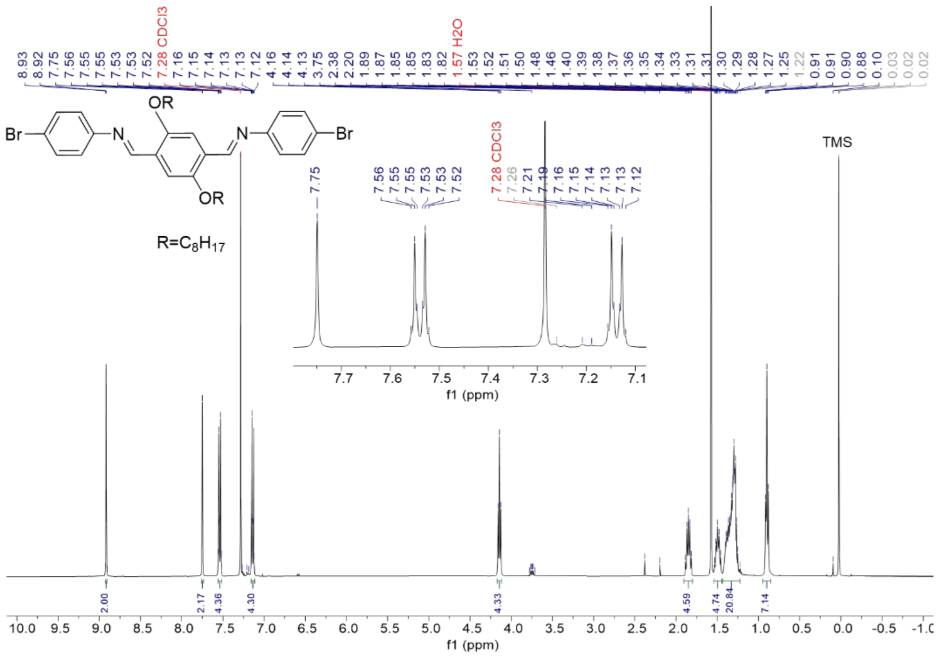

Figure S3. ^{1}H NMR (400 MHz, 25 ºC, $CDCl_3$) of Br_2Az_2.

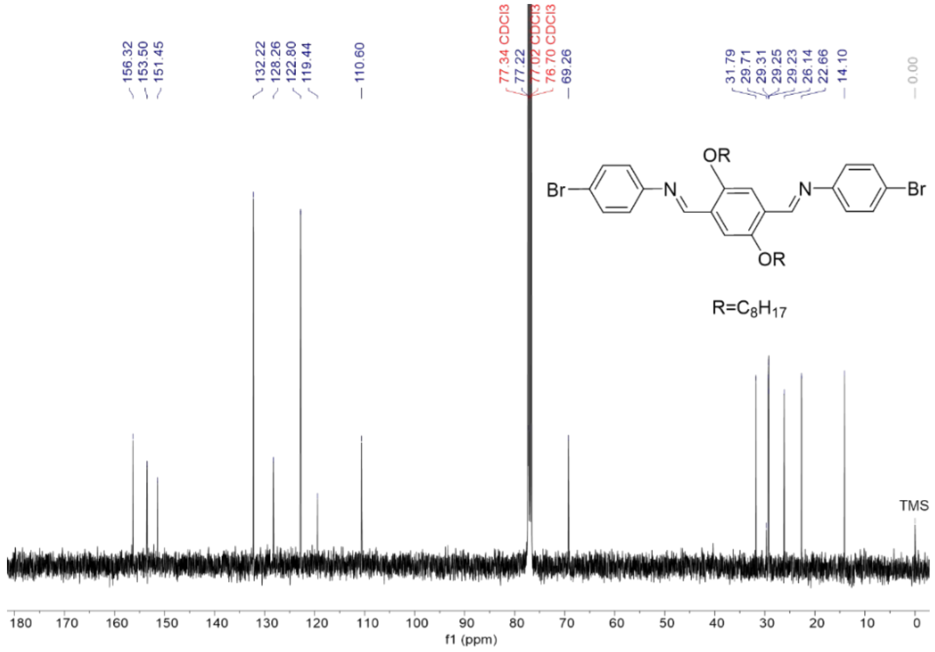

Figure S4. ^{13}C NMR (400 MHz, 25 ºC, $CDCl_3$) of Br_2Az_2.

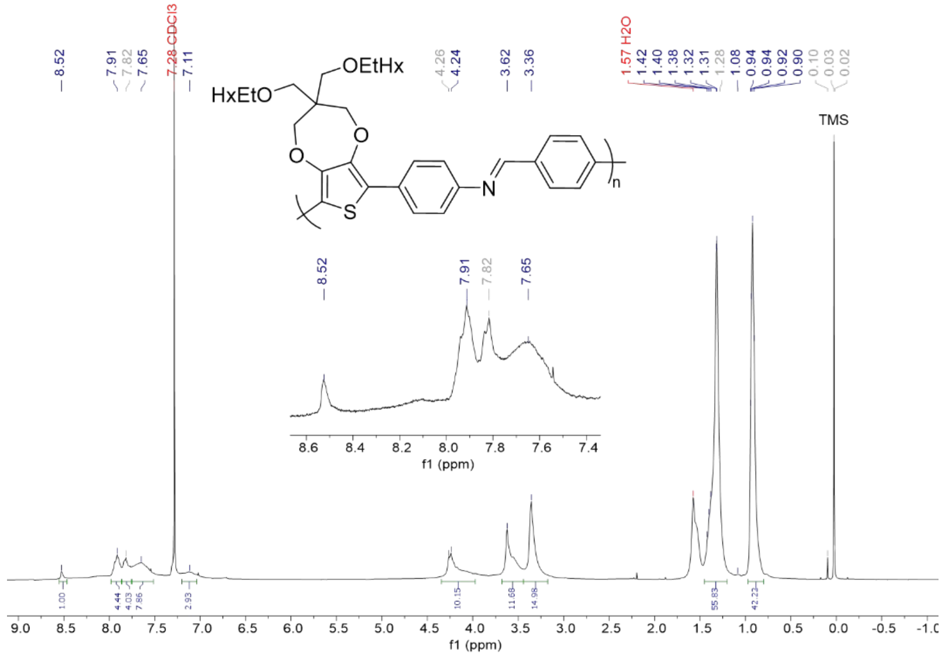

Figure S5. ^{1}H NMR (400 MHz, 25 ºC, $CDCl_3$) of polyAzProDOT.

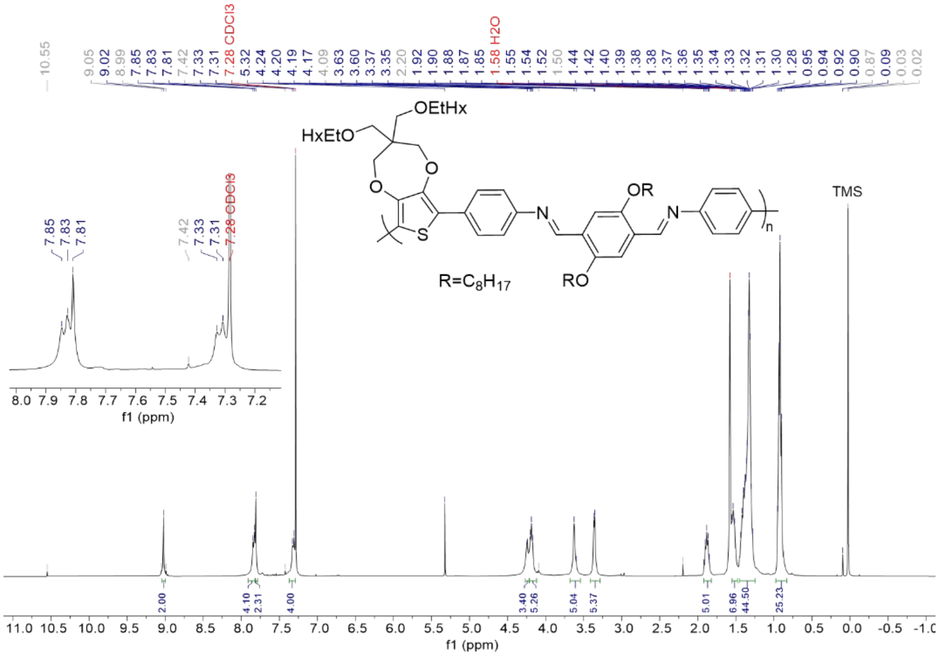

Figure S6. ^{1}H NMR (400 MHz, 25 ºC, $CDCl_3$) of polyAz$_2$ProDOT.

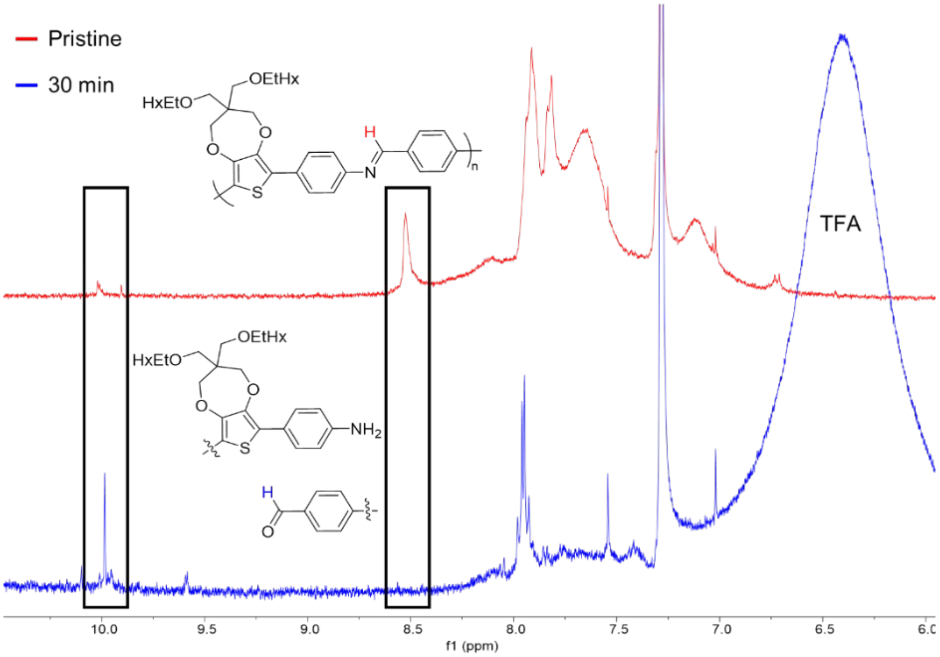

Figure S7. ^{1}H NMR of a solution of polyAzProDOT dissolved in $CDCl_3$ before (red) and 30 min after the addition of TFA (blue). The box from 8.4 ppm < δ < 8.6 ppm highlights the disappearance of the imine peak with addition of TFA and the box from 9.9 ppm < δ < 10.1 ppm highlights the increase in intensity of the aldehyde peak.

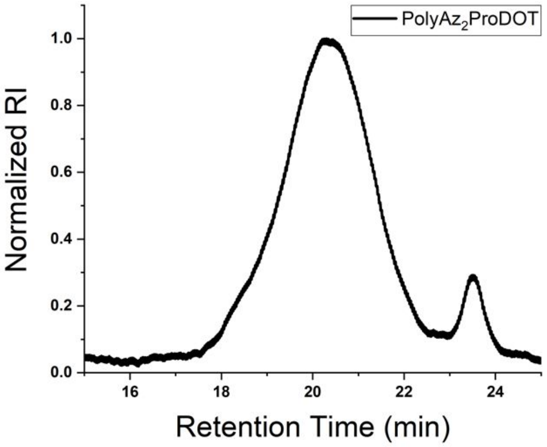

Figure S8. GPC elugram of polyAz$_2$ProDOT using trichlorobenzene as the eluent at 130 °C with a flow rate of 1 mL/min. M_n = 8.8 kg/mol, M_w/M_n = 2.1.

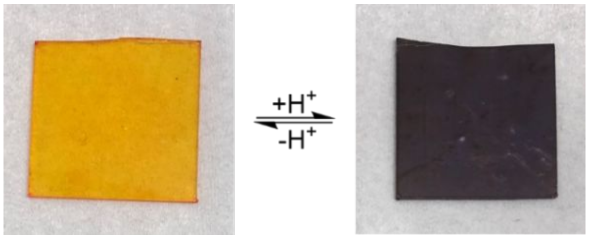

Figure S9. Photographs of a spin-coated polyAz$_2$ProDOT film before (left) and after (right) being exposed to TFA vapors.

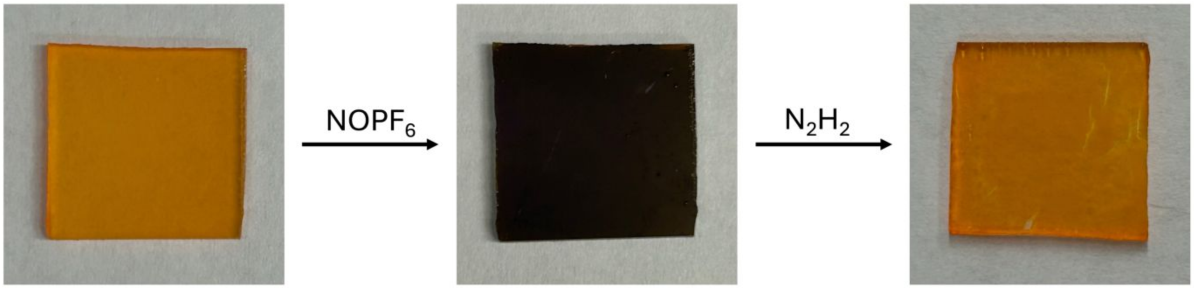

Figure S10. Photographs of a spray-coated polyAz$_2$ProDOT film prior to oxidation, after oxidative doping with $NOPF_6$, and after reduction with N_2H_2 back to the neutral film (left to right) to demonstrate the reversible chemical doping of the polymer.

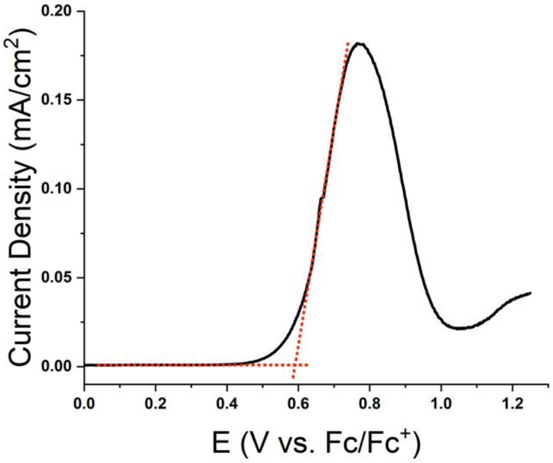

Figure S11. DPV trace for a polyAz$_2$ProDOT film spray-casted onto an ITO/glass working electrode in a 0.1 M TBAPF$_6$/ACN electrolyte solution with a step time of 0.1 s, a step size of 2 mV, and amplitude of 25 mV.

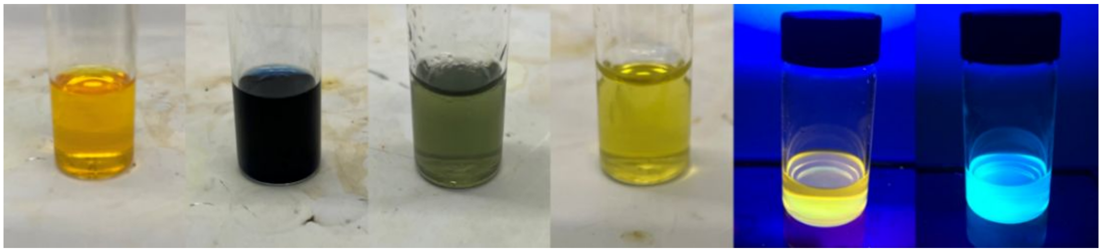

Figure S12. Photographs of polyAzProDOT in $CHCl_3$ before, immediately, 1 hour, and 24 hours after addition of TFA. Photographs of polymer solutions irradiated with UV-light before and after degradation are also shown (left to right).

UV-vis Absorbance Spectroscopy

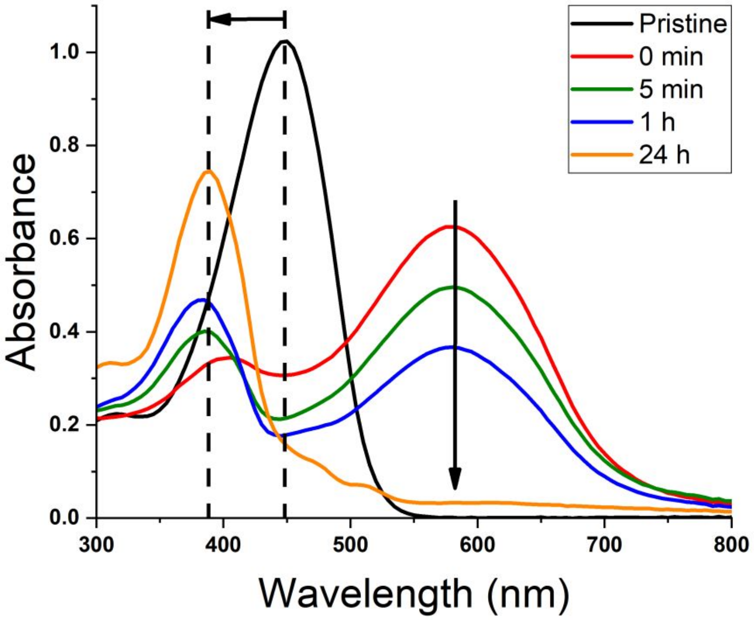

Figure S13. UV-vis absorbance spectra as a function of time of a 0.01 mg/mL solution of polyAzProDOT dissolved in $CHCl_3$ after the addition of 3 drops of a 0.4 M TFA/$CHCl_3$ solution.

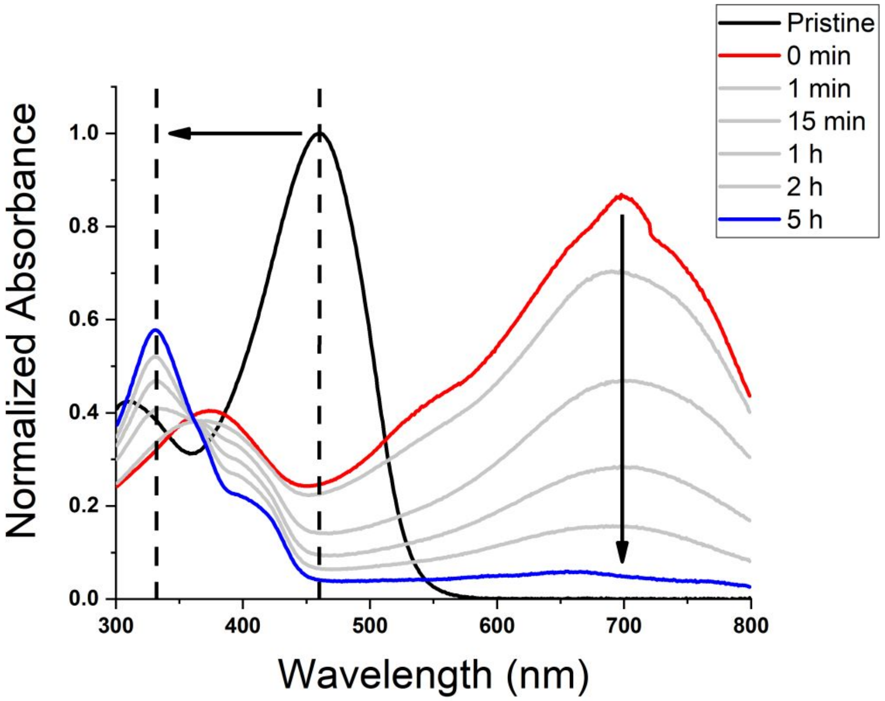

Figure S14. UV-vis absorbance spectra as a function of time of a 0.01 mg/mL solution of polyAz$_2$ProDOT dissolved in $CHCl_3$ after the addition of 50 μL of a 0.4 M TFA/$CHCl_3$ solution.

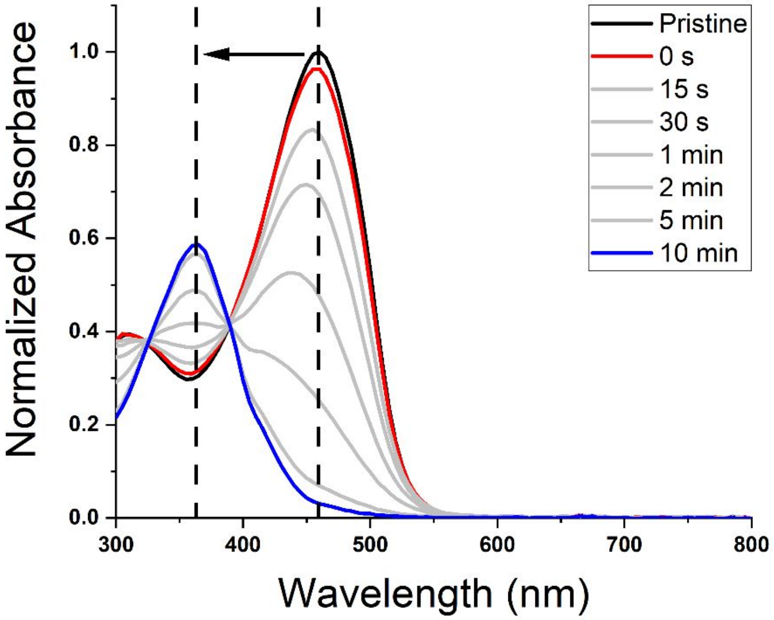

Figure S15. UV-vis absorbance spectra as a function of time of a 0.01 mg/mL solution of polyAz$_2$ProDOT dissolved in THF after the addition of 50 μL of a 0.4 M TFA/THF solution.

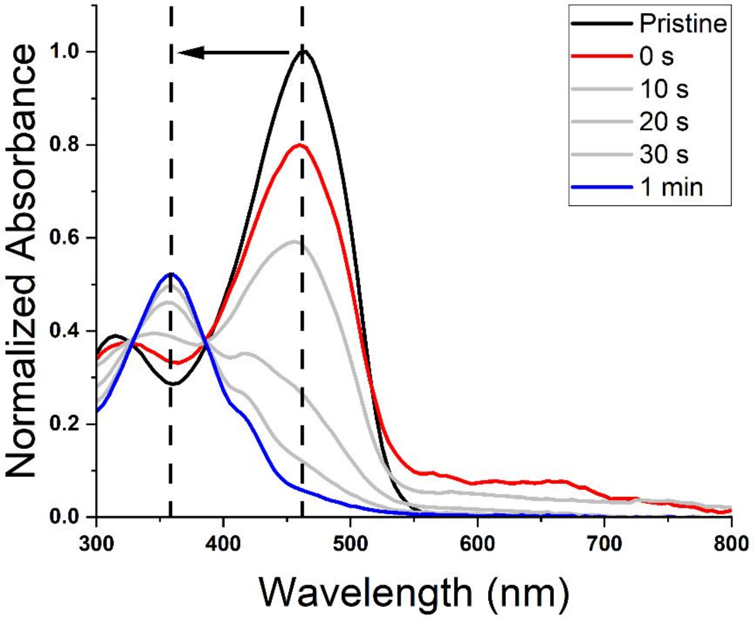

Figure S16. UV-vis absorbance spectra as a function of time of a 0.01 mg/mL solution of polyAz$_2$ProDOT dissolved in toluene after the addition of 50 μL of a 0.4 M TFA/EtOH solution.

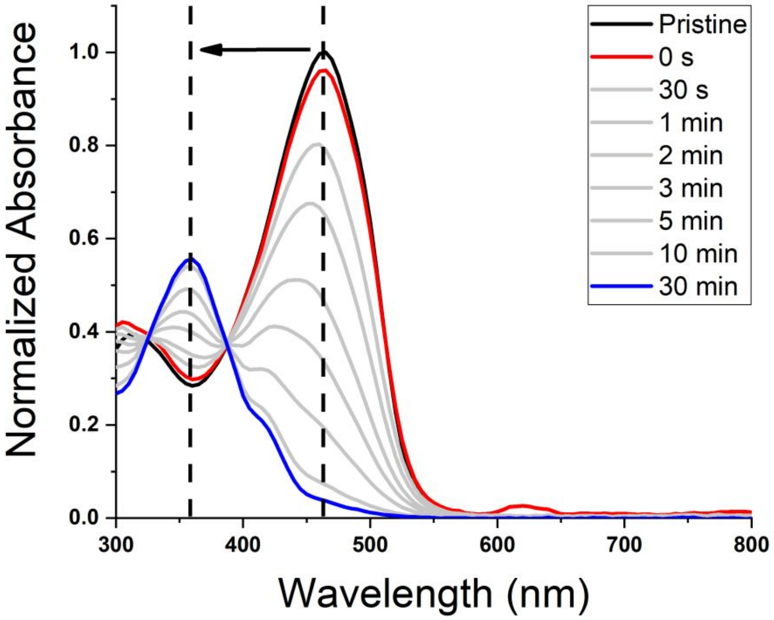

Figure S17. UV-vis absorbance spectra as a function of time of a 0.01 mg/mL solution of polyAz$_2$ProDOT dissolved in toluene after the addition of 50 μL of a 0.4 M $(COOH)_2$/EtOH solution.

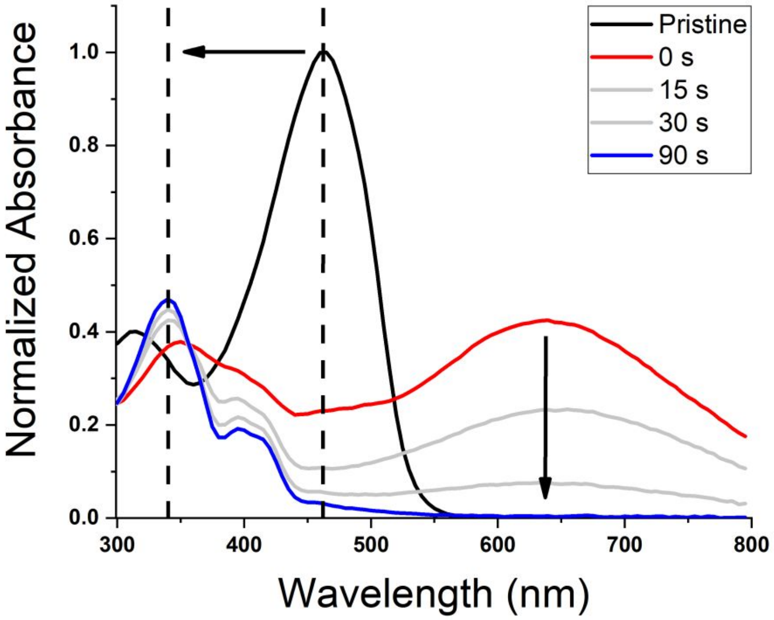

Figure S18. UV-vis absorbance spectra as a function of time of a 0.01 mg/mL solution of polyAz$_2$ProDOT dissolved in toluene after the addition of 50 μL of a 0.4 M *p*-TSA/EtOH solution.

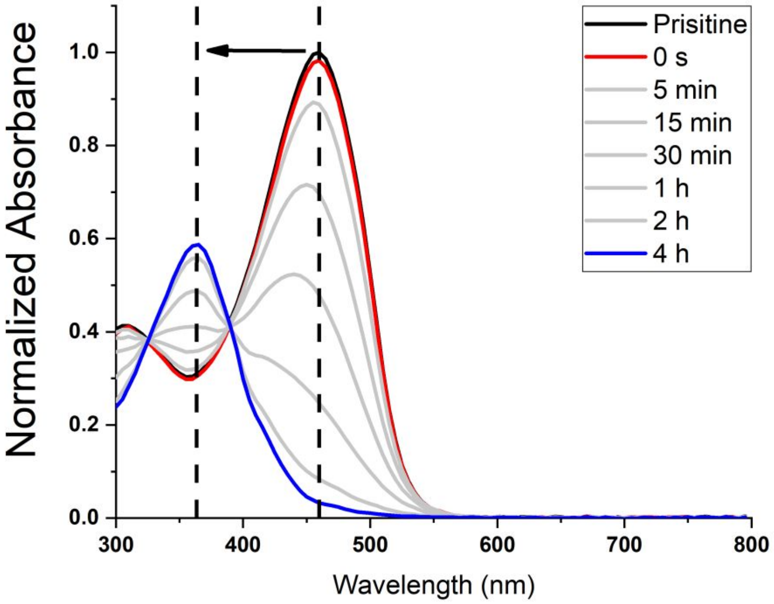

Figure S19. UV-vis absorbance spectra as a function of time of a 0.01 mg/mL solution of polyAz$_2$ProDOT dissolved in THF after the addition of 50 μL of a 0.4 M H_3PO_4/THF solution.

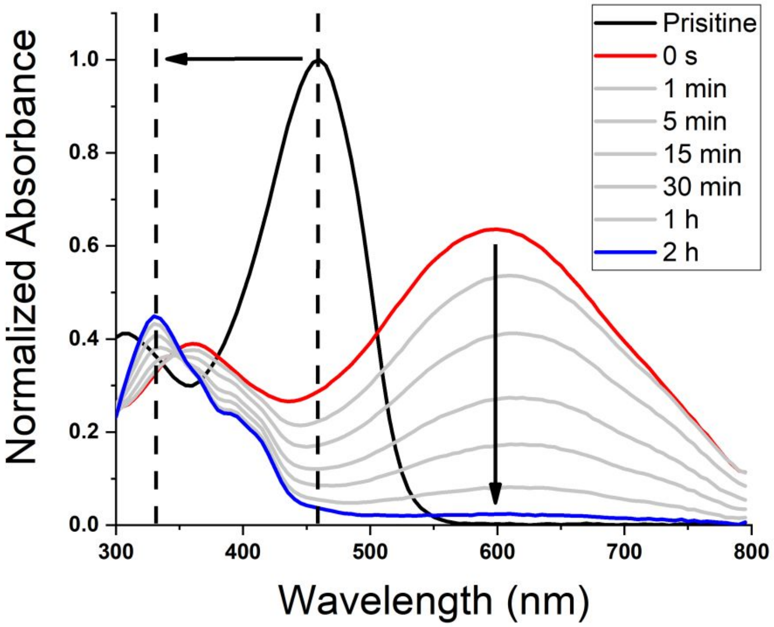

Figure S20. UV-vis absorbance spectra as a function of time of a 0.01 mg/mL solution of polyAz$_2$ProDOT dissolved in THF after the addition of 50 μL of a 0.4 M HCl/THF solution.

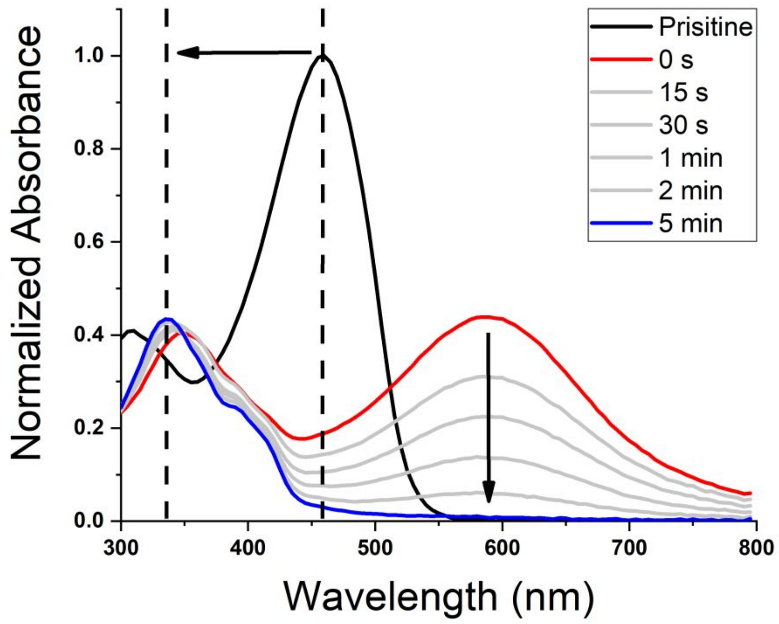

Figure S21. UV-vis absorbance spectra as a function of time of a 0.01 mg/mL solution of polyAz$_2$ProDOT dissolved in THF after the addition of 50 μL of a 0.4 M H_2SO_4/THF solution.

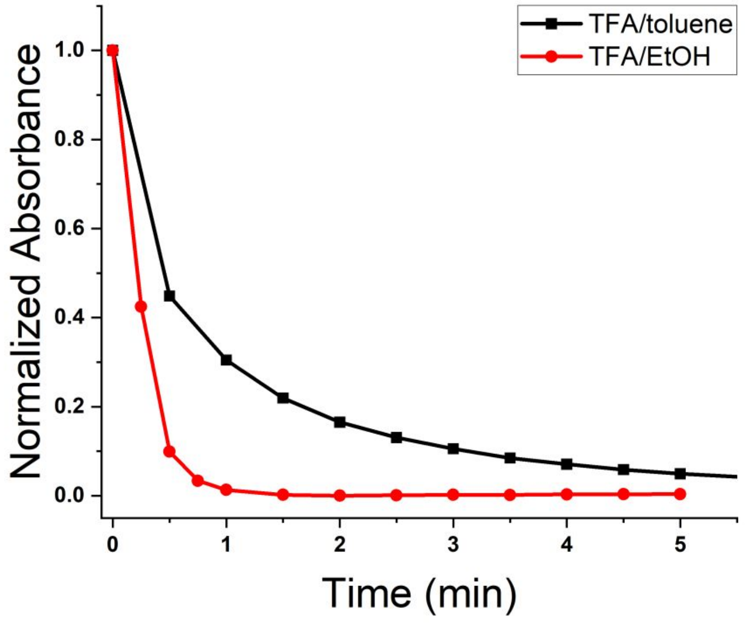

Figure S22. UV-vis absorbance at the λ_{max} as a function time for a polyAz$_2$ProDOT solution after the addition of TFA/toluene (black) or TFA/EtOH (red) stock solutions.

References

(1) Reeves, B. D.; Grenier, C. R. G.; Argun, A. A.; Cirpan, A.; McCarley, T. D.; Reynolds, J. R. Spray Coatable Electrochromic Dioxythiophene Polymers with High Coloration Efficiencies. *Macromolecules* **2004**, *37* (20), 7559–7569. https://doi.org/10.1021/MA049222Y.

(2) Lei, T.; Chen, X.; Pitner, G.; Wong, H. S. P.; Bao, Z. Removable and Recyclable Conjugated Polymers for Highly Selective and High-Yield Dispersion and Release of Low-Cost Carbon Nanotubes. *J. Am. Chem. Soc.* **2016**, *138* (3), 802–805. https://doi.org/10.1021/JACS.5B12797.
